# Oral health care access and utilization among people with hearing impairments: evidence from a Jordanian comparative study

**DOI:** 10.3389/fresc.2026.1757495

**Published:** 2026-07-09

**Authors:** Sabha Mahmoud Alshatrat, Wael Mousa Al-Omari, Abedelmalek Kalefh Tabnjh, Majd M. Alsaleh, Siddharthan Selvaraj, Zaineh W. Alomari

**Affiliations:** 1 Department of Applied Dental Sciences, College of Applied Medical Sciences, Jordan University of Science and Technology, Irbid, Jordan; 2 Department of Prosthodontics, Faculty of Dentistry, Jordan University of Science and Technology, Irbid, Jordan; 3 Department of Cariology, Odontology School, Sahlgrenska Academy, Gothenburg University, Gothenburg, Sweden; 4 Department of Pathology, Saveetha Medical College and Hospital, Saveetha Institute of Medical and Technical Sciences, Chennai, India; 5 Department of Pediatric Dentistry, College of Dentistry, University of Illinois Chicago, Chicago, IL, United States; 6 Faculty of Dentistry, University of Puthisastra, Phnom Penh, Cambodia; 7 Department of Dental Research Cell, Dr.D.Y. Patil Dental College & Hospital, Pune, India; 8 Faculty of Dentistry, Jordan University of Science and Technology, Irbid, Jordan

**Keywords:** access to dental care, barriers to dental care, deaf, hearing impairments, Jordan

## Abstract

**Background:**

Oral health is a vital component of overall well-being; however, individuals with hearing impairments often experience substantial challenges in accessing appropriate dental care. In Jordan, limited research has explored oral health disparities among individuals with hearing impairments, emphasizing the need for a focused investigation.

**Objective:**

This study aimed to assess the utilization of dental services among individuals with hearing impairments in Jordan and to identify key barriers affecting their access to dental care, in comparison with individuals without hearing impairments.

**Methods:**

A cross-sectional comparative study was conducted involving 289 participants, including 140 individuals with hearing impairments and 149 individuals without hearing impairments. Data on dental service utilization, reasons for dental visits, and perceived barriers to accessing care were collected using a validated, self-administered, closed-ended questionnaire. Participants were recruited through convenience sampling from centers and associations supporting individuals with hearing impairments. Data analysis was performed using SPSS® version 22, with statistical significance set at *p* < 0.05. Group differences were examined using Chi-square tests and contingency table analyses.

**Results:**

Participants with hearing impairments reported fewer dental appointments in the past year compared to controls (47% vs. 64%, respectively; *p* < 0.05). Toothache was the most common reason for the most recent dental visit in both groups (60.8% vs. 62.4%). Participants with hearing impairments reported significantly more barriers to dental care than controls. These included distance to dental offices (25% vs. 12.8%; *p* = 0.008), inaccessible parking (14.3% vs. 6.0%; *p* = 0.020), inadequate dental facilities (14.3% vs. 3.4%; *p* = 0.001), lack of dentist knowledge about treating individuals with disabilities (16.4% vs. 6.1%; *p* = 0.005), limited availability of specialists (18.6% vs. 9.4%; *p* = 0.024), and embarrassment (26.8% vs. 9.4%; *p* < 0.001.

**Conclusion:**

This study demonstrated socioeconomic disparities in dental service utilization among individuals with hearing impairments, alongside multiple barriers to accessing care. The findings highlight the most commonly perceived obstacles to oral health services within this population and provide valuable insights to guide the development of targeted health policies and interventions aimed at improving access to dental care and reducing oral health inequalities within this vulnerable population.

## Introduction

Oral health is a crucial component of a person's overall well-being, having a profound impact on an individual's quality of life. Although dental care services have improved significantly worldwide, inequities persist in access to and receipt of dental care services, particularly among disadvantaged and underrepresented populations. Individuals with hearing impairments, who have profound hearing loss and usually communicate using sign language, are amongst the most disadvantaged populations in access to and timely receipt of dental care services (1). Barriers to access may often arise from a complex interplay of factors, including communication barriers, inadequate awareness among dental providers, socioeconomic disadvantages, and a lack of accountability within the broader healthcare system. Globally, evidence shows that individuals with disabilities, including those who have hearing impairment, experience poorer oral health outcomes and lower utilization of dental services compared with the general population. The lack of access can typically be summed up in factors such as a lack of appropriate culturally and linguistically sensitive services, provider training to support disability competence, and societal stigma. For individuals with hearing impairment, communication between the patient and the dental professional is especially crucial (2).

The use of verbal direction and explanation has historically led to misunderstandings, anxiety, inadequate treatment, and failure to obtain informed consent in dental care. The scarcity of qualified interpreters or accessible communication technology often leaves patients with hearing impairment feeling isolated from the healthcare experience (3–5).

This issue is particularly concerning in Jordan, a middle-income country with an evolving healthcare infrastructure. Despite ongoing developments with accessibility and care quality, the Jordanian health system continues to struggle with supporting those with special needs. The hearing impairment community in Jordan is estimated to include thousands, many of whom are socially, educationally, and economically marginalized (6–8). Although governmental and non-governmental initiatives in Jordan provide some support for individuals with disabilities, there is a notable lack of research and epidemiological data addressing the specific healthcare, particularly dental barriers, experienced by individuals with hearing impairments. These include challenges with communication among dental staff, who do not receive training in Jordanian Sign Language, a lack of interpreter services in clinics or hospitals, long wait times, financial constraints, and transportation difficulties, especially in rural areas (9, 10). Furthermore, there are no public health awareness campaigns in Jordan that are specifically designed to reach or engage such a community. These, along with interpersonal and structural barriers, may lead to poor treatment time suggestions, a higher burden of oral disease, or a low level of trust in healthcare systems and providers (11, 12).

Although disability-inclusive healthcare is gaining attention worldwide, there is limited academic literature on the intersection of hearing impairment and oral health in Jordan. Most existing studies on dental care have examined patterns in the general population or expanded the scope of research to include people with disabilities (13). There is a need for contextualized research that highlights the lived experiences of such individuals and examines the factors that influence their utilization of dental services. The dearth of research in this area is acutely important, as understanding healthcare access through evidence-based practices and contextually appropriate interventions is necessary to promote equitable access (14, 15). This research will contribute to the existing literature by exploring the patterns of dental service use among individuals with hearing impairment in Jordan, as well as the access barriers they face, and the socio-cultural and institutional factors that shape their experience. The findings of this study are expected to provide a comprehensive understanding of the barriers faced by this marginalized population. The goal is for the results to inform health policy and dental practice in Jordan, promoting approaches that are more inclusive, equitable, and responsive to the needs of these individuals, as well as those of other marginalized populations more broadly.

## Materials and methods

This study was conducted in full accordance with the ethical principles outlined in the Declaration of Helsinki of the World Medical Association. The study protocol was approved by the Institutional Review Board of Jordan University of Science and Technology (Reference: 2017/0032). A cross-sectional survey was carried out to identify barriers to accessing oral healthcare among individuals with hearing impairments in Jordan. A self-designed Arabic questionnaire was developed to ensure cultural and linguistic appropriateness for participants in Jordan. Content validity was assessed by two expert panels, yielding an average congruency percentage (ACP) of 92%, which indicated a high level of agreement regarding the relevance and suitability of the items. Reliability was evaluated using a test–retest procedure with 10 individuals with hearing impairments, yielding a Cronbach's alpha coefficient of 0.75, indicating acceptable internal consistency. A pilot study involving 10 participants with hearing impairments (not included in the final sample) was also undertaken to assess the clarity, content, and format of the questionnaire. Feedback obtained from the pilot study was incorporated into the final version of the study. For younger participants, caregivers were permitted to assist with reading or explaining the questions to ensure comprehension, while responses were intended to reflect the participants' own experiences. The finalized questionnaire comprised three sections: five items assessing participants' and caregivers' demographic information; thirteen items addressing barriers to dental care, adapted and modified from previously published studies (16–18); and five items evaluating dental service utilization. The severity of hearing impairment among participants was obtained from official records provided by center managers, based on professional audiological evaluations documented in participants' files, rather than self-reports. These data were collected for descriptive purposes only and were not included as analytical variables in the statistical comparisons A list of centers and associations serving individuals with hearing impairments was obtained from the Ministries of Social Development and Education. Participants were recruited through convenience sampling at centers across Jordan's northern, central, and southern regions, which may have introduced selection bias. The inclusion of these centers was based on administrators' willingness to facilitate the distribution of questionnaires to potential participants. At the participating centers, paper-based questionnaires were distributed to participants. A cover letter accompanied the questionnaire, explaining the purpose of the study, emphasizing voluntary participation, and assuring confidentiality of the responses. The comparison group, comprising individuals without hearing impairments, was recruited from the same geographic areas through convenience sampling at schools, shopping malls, and public parks, which may have introduced selection bias. Participants with hearing impairments were eligible for inclusion if they met the following criteria: (1) a diagnosis of hearing impairment confirmed by official audiological records, (2) age 6 years or older to capture experiences from school age onwards, (3) ability to communicate in Arabic, (4) willingness to participate in the study, and (5) registration at or attendance of a center or association serving individuals with hearing impairments in Jordan. Individuals without hearing impairments were eligible for inclusion if they met the following criteria: (1) no history of hearing impairment or other significant communication disorders, (2) age within a comparable range to the hearing-impaired group, (3) ability to communicate in Arabic, and (4) willingness to participate in the study.

Questionnaires were collected within one to three weeks of distribution. For non-respondents, a reminder note was sent after three weeks to encourage participation. Overall, data collection was conducted over a six-month period. To ensure ethical compliance, a written informed consent form was provided along with the questionnaire in accordance with the IRB-approved protocol. Participants were instructed to read, sign, and return the consent form before completing the questionnaire if they agreed to participate in the study. For participants under 18 years of age, written consent was obtained from parents or legal guardians, while adult participants provided consent independently. Study staff and caregivers were available to clarify the study procedures and questionnaire items when needed. All study information was presented in simple Arabic to ensure comprehension.

Matching, where possible, was performed by insurance status and gender, and the participants' age ranges were kept as comparable as possible to minimize demographic differences between groups.

The sample size was calculated using G*Power software, based on the study by Alshatrat et al. (2024) (18), with a statistical power of 80% and a 5% margin of error. The minimum required sample size was determined to be 139 participants per group. To account for potential nonresponse, 200 questionnaires were distributed to each group. Of these, 140 were returned by individuals with hearing impairments, and 149 were returned by individuals without hearing impairments. Informed written consent was obtained along with the questionnaire. Data were analyzed using IBM SPSS Statistics version 25 (IBM Corp., NY, USA) with guidance from a biostatistician. Descriptive statistics were calculated for continuous variables (mean ± standard deviation) and categorical variables (frequency and percentage). Associations between categorical variables were assessed using Pearson's Chi-square test, with statistical significance set at *p* < 0.05.

## Results

This study involved 289 participants, including 140 with hearing impairments and 149 without. In the comparison group (without hearing impairment), ages ranged from 7 to 75 years (average age: 23), while participants with hearing impairments ranged from 7 to 61 years (average age: 15). The gender distribution in the hearing impairment group was 52% male and 48% female, compared to 67% male and 32% female in the non–hearing impairment group. Over 90% of participants in both groups were single.

The sociodemographic characteristics of the participants are summarized in Table 1. Notable differences were observed between the two groups in several demographic variables. Participants with hearing impairments were generally younger, with 87.9% under 18 years old, compared to 65% in the control group. Differences were also seen in family income distribution. More than half of the participants with hearing impairments (50.7%) reported a family income of less than 250 JD, while only 1.3% of participants without hearing impairments fell into this income category. Conversely, higher income levels were more frequently reported among the control group, with 43% reporting a family income between 500 and 1,000 JD, compared to 2.9% in the hearing impairment group.

**Table 1:** Sociodemographic characteristics of participants with and without hearing impairment groups.

Characteristics	Hearing impairment group		Without hearing impairment group	
	*n*	(%)	*n*	(%)
Family income (JD)				
<250	71	(50.7%)	2	(1.3%)
250–500	63	(45.0%)	70	(47.0%)
500–1,000	4	(2.9%)	64	(43.0%)
>1,000	2	(1.4%)	13	(8.7%)
Age				
<18	123	(87.9%)	97	(65%)
18–40	13	(9.3%)	37	(25%)
>40	4	(2.9%)	15	(10%)
Insurance				
Yes	95	(67.9%)	100	(67.1%)
No	45	(32.1%)	49	(32.9%)
Disability center/region				
Amman	131	(93.6%)	55	(36.9%)
North	6	(4.3%)	68	(45.6%)
South	2	(1.4%)	26	(17.4%)
Severity of disability				
None	0	(0%)	149	(100%)
Mild	14	(10.0%)	0	(0%)
Moderate	46	(32.9%)	0	(0%)
Severe	80	(57.1%)	0	(0%)

Insurance coverage was similar across groups, with 67.9% of participants with hearing impairments and 67.1% of participants without hearing impairments reporting having insurance. Among those with hearing impairments, the majority (93.6%) were recruited from centers in Amman, whereas participants in the control group were more geographically dispersed across northern (45.6%) and southern (17.4%) regions. Concerning hearing impairment severity, 57.1% of participants had severe impairment, 32.9% had moderate impairment, and 10% had mild impairment.

A statistically significant difference was found in the frequency of dental visits in the past year, with people with hearing impairments reporting fewer visits compared to those without hearing impairments (47% vs. 64%, respectively; *p* < 0.05).

**Figure 1:**
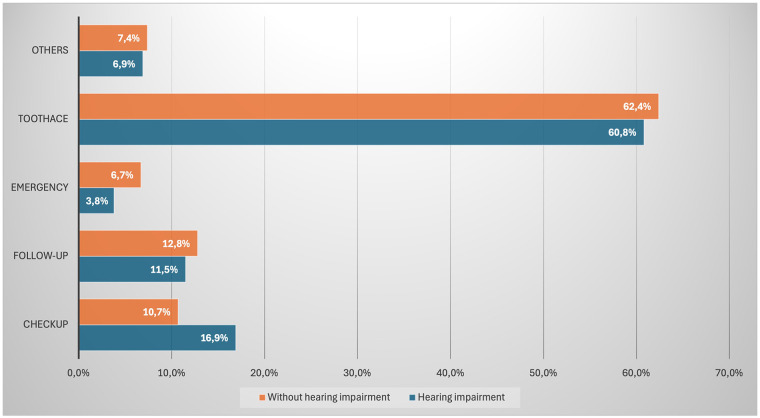
Reasons for last visit to dental service for individuals with and without hearing impairment.

The reasons for the most recent dental visit are shown in Figure 1. Toothache was the most common reason for visiting the dentist in both groups, reported by 60.8% of participants with hearing impairments and 62.4% of participants without hearing impairments. Preventive visits for routine checkups were reported by 16.9% of participants with hearing impairments and 10.7% of controls, while follow-up visits accounted for 11.5% and 12.8%, respectively. Emergency visits were the least common reason for seeking dental care in both groups, with 3.8% among participants with hearing impairments and 6.7% among controls. No statistically significant differences were found between the two groups regarding their reasons for the most recent dental appointment (*p* > 0.05).

The barriers reported when accessing dental care are shown in Table 2. Participants with hearing impairments were significantly more likely than controls to report several barriers to dental care. These included distance to dental offices (25% vs. 12.8%; *p* = 0.008), inaccessible parking areas (14.3% vs. 6.0%; *p* = 0.020), inadequate dental facilities (14.3% vs. 3.4%; *p* = 0.001), lack of dentist knowledge about treating individuals with disabilities (16.4% vs. 6.1%; *p* = 0.005), availability of only general dentists instead of specialists (18.6% vs. 9.4%; *p* = 0.024), and psychological barriers such as embarrassment (26.8% vs. 9.4%; *p* < 0.001).

**Table 2:** Barriers to dental care among participants with and without hearing impairment groups.

Barrier	Hearing impairment *n* (%)	Without hearing impairment *n* (%)	*P* value
Could not afford the cost	67 (47.9)	57 (38.3)	0.122
Dental office is too far away	35 (25)	19 (12.8)	0.008^*^
Dental office is not open at convenient times	50 (35.7)	40 (26.8)	0.104
Dental office has inaccessible parking areas	20 (14.3)	9 (6.0)	0.020^*^
Dental office has inadequate facilities to provide dental care	20 (14.3)	5 (3.4)	0.001^*^
Dentists' lack of knowledge of how to treat people with disability	23 (16.4)	9 (6.1)	0.005^*^
Dental office has a general dentist, not a specialist	26 (18.6)	14 (9.4)	0.024^*^
Long waiting time	68 (48.6)	62 (41.6)	0.235
Fear of dental work	66 (47.1)	77 (51.7)	0.441
No insurance coverage/dental coverage	52 (37.1)	52 (34.9)	0.691
Embarrassment or any psychological barriers	37 (26.8)	14 (9.4)	0.000^*^

^*^ Signiﬁcant difference, *p* < 0*.*05.

In contrast, there were no statistically significant differences between the groups in treatment cost, inconvenient clinic hours, long waiting times, dental fear, or lack of insurance coverage (*p* > 0.05).

## Discussion

Hearing impairment is one of the most common sensorineural disorders worldwide. South Asia has the highest prevalence of hearing loss, while the Middle East reports a lower prevalence of about 3%, with slightly higher rates among males (56%) compared to females (44%) (19). In Jordan, hearing impairment has been estimated at roughly 0.06% among individuals aged 12 years and older (20). Despite relatively low national estimates, the global burden of hearing loss is rising quickly, with projections indicating that 1 in 4 people could experience some degree of hearing loss by 2050 (19).

Previous research has shown that children with hearing impairments experience higher rates of dental caries, gingival inflammation, and dental trauma compared to children without hearing impairments, which can negatively impact their quality of life (21). Similar findings have been reported among hearing-impaired adults in Saudi Arabia, where higher caries experience and poorer oral health-related quality of life were documented (22). These findings highlight the ongoing unmet oral health needs among individuals with hearing impairments, partly due to barriers in accessing oral healthcare services (9). In the present study, most participants in both groups reported visiting the dentist mainly due to toothache, indicating symptom-driven dental visits rather than preventive care. Participants without hearing impairments reported more dental visits in the past year, suggesting that preventive dental care may be less accessible or less prioritized among individuals with hearing impairments. Similar patterns have been seen in earlier studies where pain was the main reason for dental visits among people with hearing impairments (13, 23). This pattern highlights the urgent need to increase public awareness about preventive dentistry and to emphasize the crucial role of maintaining oral health, particularly among individuals with disabilities, who may face additional challenges in accessing and utilizing routine dental care.

Several structural and social barriers to accessing dental care were identified. These included challenges with accessibility, communication issues, and provider-related factors, which have also been reported in earlier studies involving individuals with disabilities (9, 24, 25). Although treatment cost is often cited as a barrier to dental care (9), the difference between groups in this study was not statistically significant. However, nearly half of the participants with hearing impairments reported cost as a barrier, possibly reflecting broader socioeconomic disadvantages faced by this group, such as lower income and fewer educational opportunities (26). Socioeconomic status remains a key factor influencing oral health outcomes among individuals with disabilities, even when insurance coverage is available (27, 28).

The present findings also indicate notable socioeconomic disparities between the two groups. As shown in Table 1, approximately 50.7% of participants with hearing impairments reported a family income below 250 JD, compared with only 1.3% in the control group. In contrast, higher income categories were substantially more represented among participants without hearing impairments, with 43% reporting income between 500 and 1,000 JD. These differences suggest that individuals with hearing impairments may experience greater socioeconomic disadvantage, which could further contribute to reduced access to dental care and lower utilization of preventive dental services.

Participants with hearing impairments also noted barriers related to physical accessibility, such as the distance to dental clinics and limited parking options. These barriers may partly result from the greater reliance of younger individuals with hearing impairments on caregivers for transportation. People with sensory disabilities often face limited access to healthcare and higher risks of unmet oral health needs due to logistical and structural challenges (29).

Provider-related barriers were also identified, especially the limited availability of dentists with proper training to treat patients with special healthcare needs. Communication barriers remain a key contributor to healthcare disparities among individuals with hearing impairments (30). Previous research has also shown that many dentists feel inadequately prepared to care for patients with special healthcare needs due to limited training during their education (31–33). Insufficient training can lead to reduced accessibility, communication challenges, and lower patient satisfaction during clinical visits (2).

Participants highlighted the significance of provider competence and specialized care when treating individuals with hearing impairments. Training programs that focus on communication strategies and disability-inclusive care have been shown to enhance interactions with deaf patients and strengthen the patient-provider relationship (2). Participants also reported psychological barriers such as embarrassment and discomfort during dental visits. These experiences may reflect communication challenges and broader social issues faced by individuals with hearing impairments (7, 34). Research further suggests that deaf children may face higher levels of psychological vulnerability compared with hearing children, underscoring the need for supportive and accessible healthcare environments (35).

These findings have significant implications for policy and dental education. Dental curricula should include training on disability-inclusive care, covering communication techniques, cultural competence, and managing patients with hearing impairments. Educational components like sign language training, interpreter use, and visual communication tools could enhance communication and lower barriers to dental care. Evidence indicates that better communication between healthcare providers and patients increases trust, patient satisfaction, and healthcare utilization (36). Furthermore, multimedia educational resources tailored for individuals with hearing impairments may improve oral health education and communication in clinical settings (37).

Several limitations should be considered when interpreting the findings of this study. First, there was an age mismatch between the hearing-impaired and control groups, with more participants under 18 years old in the hearing-impaired group. This imbalance may have introduced age as a confounding factor, as age-related differences could have influenced the observed outcomes independently of hearing status. Consequently, the lack of statistical adjustment for age may limit the ability to attribute the findings solely to hearing impairment. Second, responses from younger participants may have been affected by literacy limitations, possibly introducing response bias. Third, caregiver assistance might have influenced responses among some children, potentially affecting the accuracy of self-reported information. Additionally, participants with hearing impairments were recruited from specialized centers, which could limit the generalizability of the findings to individuals with hearing impairments living outside institutional or support-center settings. Although information on the severity of hearing impairment was obtained from official records, it was not included as an analytical variable in the assessment of barriers to dental care utilization in this study. Incorporating the severity of hearing impairment into the analysis could provide valuable insights into whether individuals with more severe hearing loss experience greater challenges in accessing dental healthcare services. Future research should consider examining the relationship between the severity of hearing impairment and patterns of dental healthcare utilization to better understand the factors influencing access to oral health services among individuals with hearing impairments. In the present study, Pearson's Chi-square test was used to assess associations between categorical variables, and the assumptions of the test were examined before analysis. We acknowledge that more advanced statistical approaches, such as logistic regression, could provide further insight into predictors of barriers to dental care and allow adjustment for potential confounders such as age.

Despite these limitations, this study offers valuable insights into oral healthcare access among individuals with hearing impairments in Jordan. To our knowledge, this is the first study in Jordan to specifically explore barriers to dental care utilization in this population. The findings are important for public health planning and disability policy development and emphasize the need for targeted strategies to reduce oral health disparities among those with hearing impairments.

## Data Availability

The original contributions presented in the study are included in the article/Supplementary Material, further inquiries can be directed to the corresponding authors.
